# Flexoelectric Polarization in a Nematic Liquid Crystal Enhanced by Dopants with Different Molecular Shape Polarities

**DOI:** 10.1021/acsomega.2c00023

**Published:** 2022-03-10

**Authors:** Miha Škarabot, Nigel J. Mottram, Supreet Kaur, Corrie T. Imrie, Ewan Forsyth, John M. D. Storey, Rafal Mazur, Wiktor Piecek, Lachezar Komitov

**Affiliations:** † Condensed Matter Department, J. Stefan Institute, Jamova 39, SI-1000 Ljubljana, Slovenia; ‡ School of Mathematics and Statistics, University of Glasgow, University Place, Glasgow G12 8QQ, Scotland, U.K.; § Indian Institute of Science Education and Research (IISER) Mohali, Sector-81, Knowledge City, Manauli 140306, India; ∥ School of Natural and Computing Sciences, University of Aberdeen, Meston Building, Aberdeen A24 3UE, Scotland, U.K.; ⊥ Military University of Technology, Kaliskiego St. 2, 00-908 Warszawa, Poland; # University of Gothenburg, SE-41296 Gothenburg, Sweden; ¶ Innovidis AB, P.O. 3029, 40010 Gothenburg, Sweden; ∇ HighVisTec GmbH, Benkenstrasse 254C, CH 4108 Witterswill, Switzerland

## Abstract

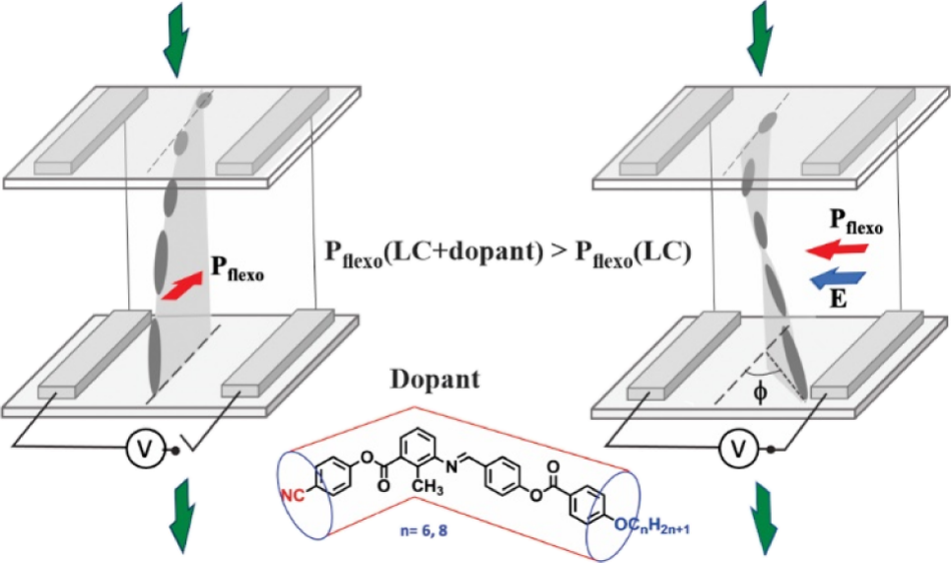

Flexoelectricity may have an important impact on the switching properties of nematic and cholesteric liquid crystals due to the linear coupling between the flexoelectric polarization of the liquid crystal and the applied electric field. This coupling is the origin of the extraordinary electro-optic effect in cholesterics aligned in the uniform lying helix texture, resulting in fast switching and field control of both rise and fall times. Therefore, the flexoelectric properties of the liquid crystals have become an important issue when designing and synthesizing liquid crystal materials and/or preparing their mixtures with appropriate flexoelectric compounds (dopants). Here, we report on the flexoelectric polarization of a highly polar nematic liquid crystal host enhanced by doping it with two newly synthesized dopants SK 1–6 and SK 1–8, possessing a hockey stick molecular shape, and comparing their doping effect with the one of the dimeric dopants CB7CB possessing a symmetric bend molecular shape. All dopants were dissolved in small concentration (5 wt %) in the nematic host so that the linear approximation of the dependence of the difference between splay *e*_s_ and bend *e*_b_ flexoelectric constants, that is, (*e*_s_ – *e*_b_), on the concentration of the dopant in the host material can be applied. In this way, (*e*_s_ – *e*_b_) was estimated for the hockey stick dopants SK 1–6 and SK 1–8 to be 0.182 and 0.204 nC/m, respectively. The obtained flexoelectric polarization of these dopants is among the highest reported in the literature so far.

## Introduction

Flexoelectricity is a well-known property of liquid crystals, analogous to the piezoelectric effect in solid materials. First described by Meyer,^1^ flexoelectricity is the induced polarization of a liquid crystal as a result of curvature strains and is usually most pronounced in materials with molecules that, in addition to a permanent dipole moment, possess “shape polarity.” The total flexoelectric polarization of the liquid crystal is given by
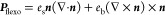
1where *e*_s_ and *e*_b_ are the flexoelectric coefficients for splay and bend elastic deformations, respectively, and ***n*** is the liquid crystal director, a unit vector defining the preferred orientation of the liquid crystal molecules.^1^

**Figure 1:**
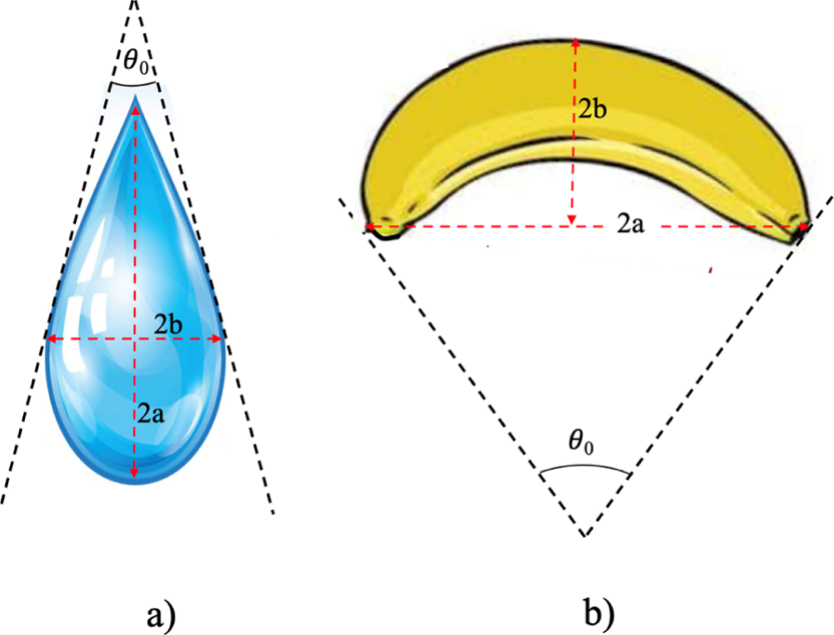
Schematic representation of the molecules with drop (a) and bend (b) shapes and their molecular parameters considered in the Helfrich model.^2^

In 1971, Helfrich^2^ derived a relatively simple dependence of the flexoelectric coefficients on the molecular shape and molecular net dipole moment (Figure 1) according to
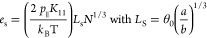
2
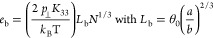
3where *p*_||_ and *p*_⊥_ are components of the molecular net dipole moments parallel and perpendicular to the molecule long axis, respectively. The degree of the molecular asymmetry (polarity) is described by the coefficients *L*_s_ for drop shape molecules and *L*_b_ for banana (bend) shape molecules, with *a* and *b* being the length and width of the molecule, respectively. The angle θ_0_ represents the opening angle for drop shape molecule and the kink angle in the bend core molecule (Figure 1). *K*_11_ and *K*_33_ are the liquid crystal splay and bend elastic constants, respectively, *N* is the number density of the molecules and *k*_B_ is the Boltzmann constant. From eqs 2 and 3, it follows that the bend flexoelectric coefficient *e*_b_ has a stronger dependence on the molecular asymmetry, represented by the ratio *a*/*b*, than the splay flexoelectric constant *e*_s_ and thus is more sensitive to changes in the molecular shape. Further work has also shown that molecular shape is of major importance for the magnitude of ***P***_flexo_(3−7) and that ***P***_flexo_ may also undergo a sign reversal.^5,8^

The control of the magnitude and direction of ***P***_flexo_ is crucial for many device applications. For instance, for display application purposes, nematic liquid crystals with large magnitude of ***P***_flexo_ were designed and synthesized (e.g., Merck, Germany).^9^ Such large values of the magnitude of ***P***_flexo_ are important for the performance of bistable nematic liquid crystal displays as well as for those having polar switching based on the linear coupling between ***P***_flexo_ and the applied electric field.^10,11^ Flexoelectric coupling also gives rise to fast electro-optic switching in short pitch cholesterics^12^ and is thus attractive for fast switching liquid crystal devices.^13^

However, the choice of such materials exhibiting high magnitude ***P***_flexo_ is very limited because most of the liquid crystals with large flexoelectric polarization usually exhibit the nematic phase only at very high temperatures and/or possess impropriate other physical properties, such as high viscosity, are difficult to align, or do not possess suitable values of dielectric anisotropy and birefringence, and so forth. To overcome these problems, methods similar to those used for tailoring the spontaneous polarization ***P***_s_ of ferroelectric liquid crystal mixtures were employed^14,15^ in order to enhance ***P***_flexo_, for instance, by dissolving an appropriate dopant in the host nematic liquid crystal.^5−7^ This method was used in the present work to evaluate the potential of the studied dopants for enhancement of flexoelectric properties of liquid crystals.

During the last two decades, numerous studies devoted to the flexoelectric properties of nematic and cholesteric liquid crystal have been undertaken and discussed.^16^ Of particular interest in this area is the discovery, more than three decades ago, that the origin of one of the most remarkable effects in liquid crystals, the electro-optic effect in cholesteric liquid crystals, is due to the coupling between ***P***_flexo_ of the cholesteric and the electric field applied perpendicular to the helix axis, when the cholesteric is aligned in uniform lying helix texture.^17,18^ Following this discovery, considerable research effort has been devoted to understanding the relationship between the liquid crystal molecular structure and the flexoelectric polarizability of the liquid crystal and has since allowed the design and synthesis of liquid crystal materials and mixtures with enhanced flexoelectric polarizability.^3−5,7,19−24^

In the continued search for dopants appropriate for tailoring the flexoelectric properties of the nematic liquid crystal host material, we investigate in this paper three possible candidate dopants with different molecular shape anisotropy, one possessing a bend molecular shape and the other two having a hockey stick molecular shape with different molecular net dipole moments and orientation. We then compare their doping effects on ***P***_flexo_ of the liquid crystal mixture they are dissolved in.

## Experimental Section

### Experimental Cells

**Figure 2:**
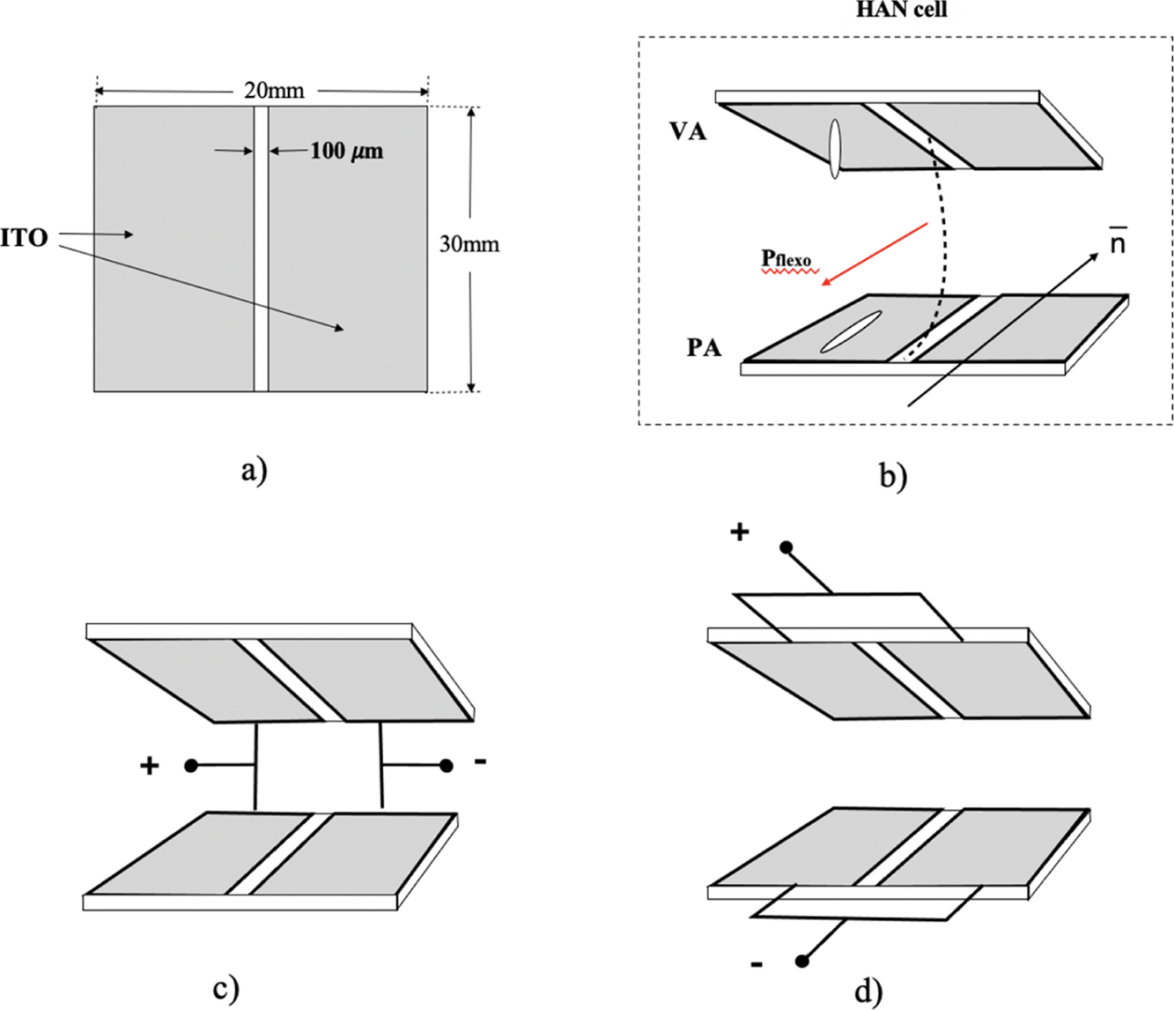
Schematic representation of the HAN experimental cell consisting of two equal substrates with a pair of electrodes deposited on their inner surface. The electrodes are separated by a slit of 100 μm (a). The substrates of the cells are assembled in such a way that the slits between electrodes deposited on the two substrates are aligned. (b) Plane of HAN is orthogonal to the substrates and parallel to the electrodes’ slit. The electrodes on both substrates are connected for generating (c) in-plane or (d) out-of-plane electric fields.

The experimental cells used in our study were of a conventional sandwich type consisting of two parallel glass plates, forming a cell with a predeterminate gap between these substrates, which is filled with liquid crystal. The liquid crystal cell gap was approximately 3–4 μm. On the inner surface of each of the cell substrates, a pair of stripe electrodes was deposited, as shown in Figure 2a, separated by a slit of 100 μm width. The inner surface of the first substrate was covered by an alignment material SE1211 (Nissan Chem, Ltd, Japan) for promoting vertical alignment of the liquid crystal molecules, whereas the inner surface of the second substrate was covered with an alignment material SE130 (Nissan Chem, Ltd, Japan), promoting planar alignment. The planar alignment layer was unidirectionally rubbed along the slit between the electrodes. As a result, the liquid crystal in the cell adopted hybrid alignment nematic (HAN) configuration with the plane of HAN configuration being orthogonal to the substrates and parallel to the electrodes’ slit, as shown in Figure 2b. The cell substrates were assembled in such way that the slits between their pair electrodes overlap each other. Such an arrangement of the substrate electrodes ensures the generation of a uniform in-plane electric field (Figure 2c), which was necessary for studying the electro-optic response of the cell under a dc applied electric field. It also enables the presence of two identical areas of the cell for studying the electro-optic response when applying an out-of-plane dc electric field (Figure 2d).

### Liquid Crystal Host and Dopants

**Figure 3:**
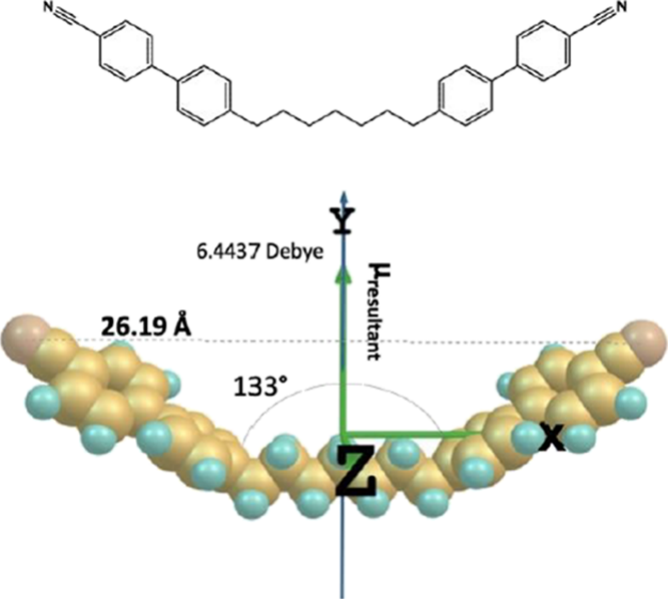
Molecular structure of CB7CB and its DFT optimized structure in a Cartesian coordinate frame, with the resultant (net) dipole moment μ_resultant_ = 6.44 Debye. CB7CB has phase sequence: NTB phase-104°C-N-17°C-Iso.

The liquid crystal materials investigated within this study were the host nematic liquid crystal MDA-09-2329 (Merck, Germany) and its mixture with the flexoelectric dopants: (a) dimer CB7CB (Figure 3), (b) SK 1–6 (Figure 5), and (c) SK 1–8 (Figure 6) dissolved in concentration of 5 wt %. For comparison, the nematic liquid crystal mixture E7 (Merck) was also studied since the physical characteristics of E7 are commonly studied by many researchers, and the necessary data for our study are available in the scientific literature.^25,26^

**Figure 4:**
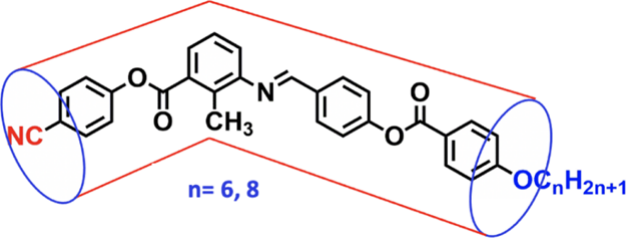
Chemical structure of the hockey-stick-shaped molecules as dopants.

**Figure 5:**
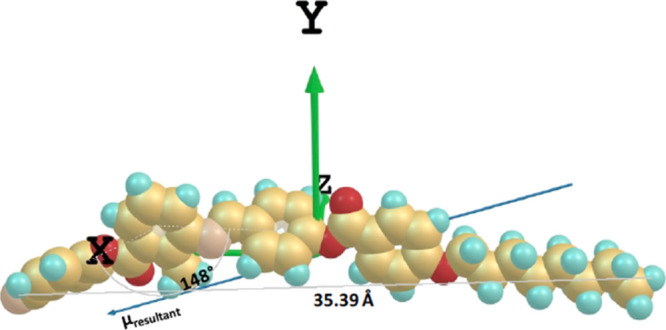
Molecular structure of SK 1–6 and its DFT optimized structure in a Cartesian coordinate frame, with the resultant (net) dipole moment μ_resultant_ = 11.34 Debye. SK 1–6 has a phase sequence of Cry-133.7°C-N-186.1°C-Iso; Iso-184.0°C-N-74.5°C-Cry.

**Figure 6:**
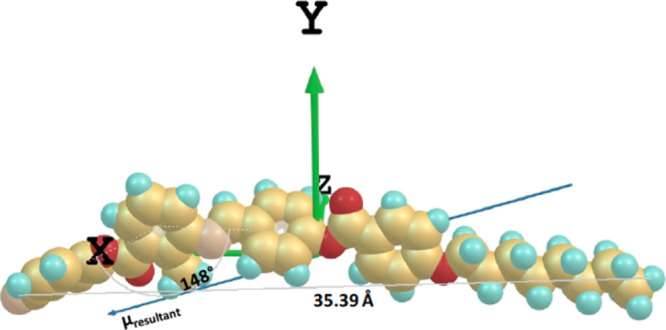
Molecular structure of SK 1–8 and its DFT optimized structure in a Cartesian coordinate frame, with the resultant (net) dipole moment μ_resultant_ = 11.22 Debye. SK 1–8 has phase sequence of Cry-122.9°C-N_cyb_-174.7°C-Iso; Iso-172.9°C-N_cyb_-86.5°C-Cry.

The synthesis and properties of the dimer CB7CB and the hockey stick molecules of SK 1–6^27,28^ and SK 1–8^29,30^ have been already reported. The last two dopants have a nematic phase at high temperatures and therefore to study their flexoelectric properties is difficult or even impossible. One of the main reasons is that it is difficult to obtain or keep the HAN configuration at such high temperatures.

Host material MDA-09-2329 (kindly supplied by Merck) is a room-temperature nematic liquid crystal mixture with transition temperature to isotropic phase at 80.5 °C and has a positive dielectric constant Δε = 21.2. In low concentrations, for example, 5 wt %, the studied dopants had good solubility in the host material. No material separation was obtained under the entire time of performing the experiments when the experimental cells were microscopically investigated.

The CB7CB molecule has a bend molecular structure with molecular net dipole moment μ_resultant_ = 6.44 Debye, as shown in Figure 3. The molecular net dipole is parallel to the *Y*-axis of the Cartesian coordinate frame due to the symmetric bend structure. The dopants SK 1–6 and SK 1–8 are hockey-stick-shaped molecules derived from 3-amino-2-methylbenzoic acid as the central core unit (Figure 4). The molecular structure is composed of four phenyl rings, where the long arm has an imine and ester linkage and the short arm has an ester linkage. One terminal is tethered with a polar −CN moiety and the other terminal with a flexible alkyl chain (*n* = 6, 8). The details of synthesis and characterization of the dopants are reported elsewhere.^29^ The dopants SK 1–6 and SK 1–8 have molecular net dipole moment μ_resultant_ = 11.34 Debye and μ_resultant_ = 11.22 Debye, respectively, as shown in Figures 5 and 6. The molecular net dipole moments, according to their DFT optimized structure, are inclined with respect to *X*-axis of the Cartesian coordinate frame, being 20.5°, for SK 1–6, and 17.8°, for SK 1–8. Moreover, the dopant SK 1–8 has a cybotactic nematic phase above the crystal phase.^29,30^ The summarized data of the optimized DFT structure of the dopants are presented in Table 1.

**Table 1:** Data from the Optimized Structure of the Dopants CB7CB, SK 1–6, and SK 1–8

CB7CB					
dipole moment, μ (Debye)					
μ_*X*_	μ_*Y*_	μ_*Z*_	μ_resultant_	bent angle, θ (deg)	molecular length *L* (Å)
0.0001	6.4366	0.3026	6.4437	133	26.19
the net dipole moment (6.44 D) is in the *Y*-direction					

compound 1–6					
dipole moment, μ (Debye)					
μ_*X*_	μ_*Y*_	μ_*Z*_	μ_resultant_	bent angle, θ (deg)	molecular length *L* (Å)
10.62	3.97	0.3	11.34	148	32.56
the net dipole moment (11.34 D) is in the *X*-direction					

compound 1–8					
dipole moment, μ (Debye)					
μ_*X*_	μ_*Y*_	μ_*Z*_	μ_resultant_	bent angle, θ (deg)	molecular length *L* (Å)
10.68	3.43	0.12	11.22	148	35.39
the net dipole moment (11.22 D) is in the *X*-direction					

### Experimental Setup

**Figure 7:**
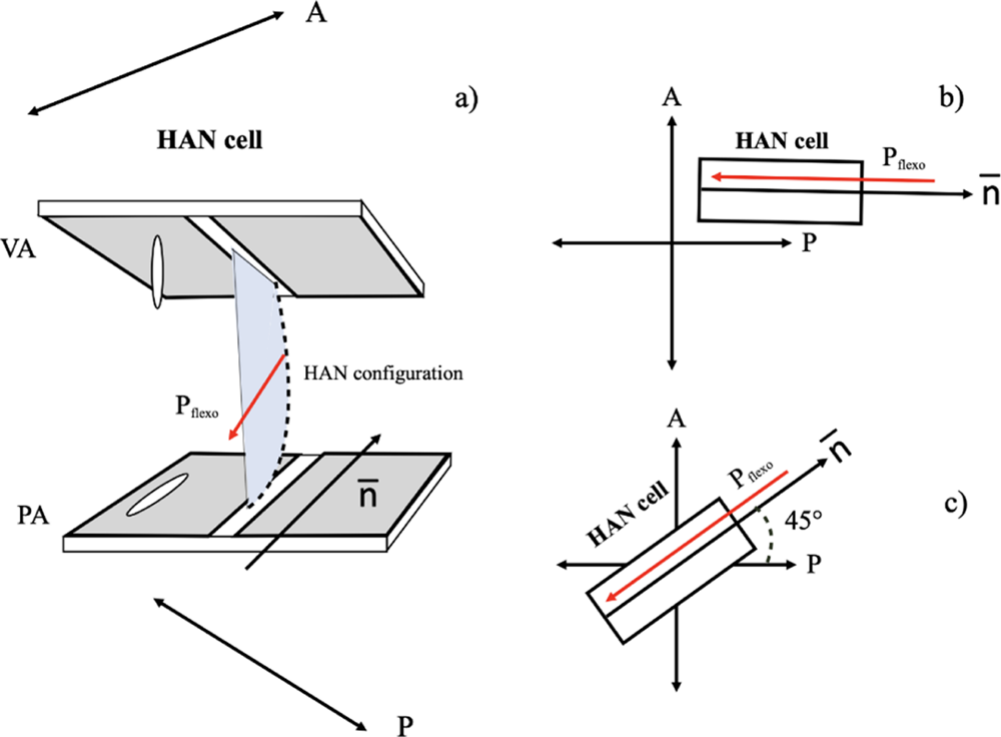
(a) HAN configuration of the liquid crystal in the experimental cells. Orientation of the HAN cell inserted between crossed linear polarizers of the polarizing microscope for measuring the light transmitted intensity as a function of the applied voltage in the case of (b) in-plane and (c) out-of-plane applied dc electric fields, respectively.

The experimental cells were placed in a Nikon Eclipse 600 Pol polarizing microscope between crossed linear polarizers, and the light transmission intensity through the experimental cells was measured using the digital camera Pixelink PLA741. A green bandpass filter (λ = 545 nm) was used to select the wavelength of the illuminating light. The cell was set with the rubbing direction of the substrate, promoting planar alignment either at 0° with respect to one of the crossed polarized, when applying in-plane applied dc electric field, or at 45° with respect to the polarizers, when applying out-of-plane electric field (see Figure 7b,c).

The voltage was applied to the cell using a function generator (Rigol DG1022Z). The electro-optical response of the experimental cells was investigated at applied in-plane and out-of-plane dc electric fields. For the measurements performed under in-plane dc electric field, the direction of the applied field was orthogonal to the HAN deformation plane (Figure 2b,c), whereas for those performed under out-of-plane dc electric field (Figure 2b,d), the applied field was directed parallel to the deformation plane of HAN configuration. For applied in-plane dc electric field (Figure 2c), the switching of the molecules took place to a large extent in the plane of the cell substrates, especially in the center of the slit between the electrodes. When the dc electric field is applied across the cell, that is, out-of-plane field (Figure 2d), the switching of the liquid crystal molecules took place substantially in the plane of HAN configuration, that is, in a plane orthogonal to the substrates and parallel to the electrodes’ slit.

The direction of the in-plane switching of the molecules was first used to measure the sign of ***P***_flexo_ of the host nematic liquid crystal and its mixtures with the dopants CB7CB, SK 1–6, and SK 1–8. The sign of flexoelectric polarization was determinate according to the original method.^18^

## Theoretical

Various methods for the measurement of *e*_s_ and *e*_b_, or equivalently (*e*_s_ – *e*_b_) and (*e*_s_ + *e*_b_), are described in the literature,^31−34^ all of which derive from the fitting of experimental data to theoretical models. In this work, we use methods for deriving (*e*_s_ – *e*_b_)^31^ and (*e*_s_ + *e*_b_).^26^

### In-plane Theoretical Model

**Figure 8:**
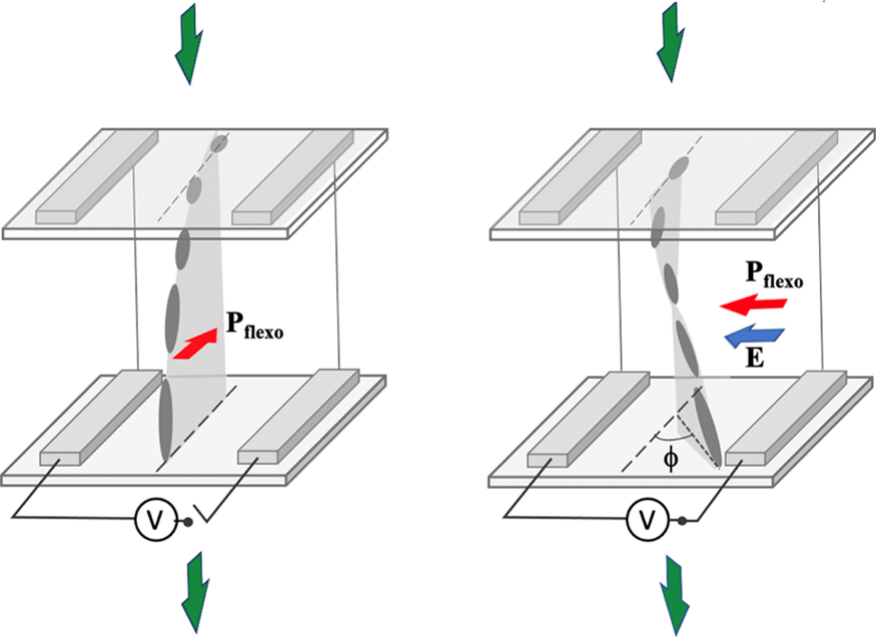
Schematic picture of the rotation of the director in the HAN configuration due to the flexoelectric coupling when an in-plane dc electric field is applied.

The difference between *e*_s_ and *e*_b_, that is, (*e*_s_ – *e*_b_), was derived from the electro-optical response arising from the in-plane switching of the liquid crystal in the HAN cell,^31^ presented in Figure 8. The applied in-plane dc electric field in the HAN cell is generated between the two pairs of electrodes connected as shown in Figure 2c. The applied in-plane dc electric field resulted in switching of the liquid crystal molecules in the plane of the cell substrates, so called in-plane switching. In this switching process, both flexoelectric and dielectric coupling (due to the positive Δε of the mixtures) are acting in the same direction, along the applied electric field. As a result, a twist configuration forms along the substrate normal due to the competition between the elastic torque, present when the director *n* moves in-plane away from the azimuthal preferred anchoring direction at the substrates, and the orienting electric field torque perpendicular to this direction (Figure 8). Although the induced twist deformation is due to both flexoelectric and dielectric coupling, at low voltages, the flexoelectric coupling plays a major role in the in-plane switching, and thus the resulting twist is linearly dependent on the applied electric field (cf. Figure 8). A simple model of this electro-optic effect, suggested by Dozov et al.,^31^ arising from flexoelectric coupling assumes that the twist of the director along the substrate normal leads to a rotation of the polarization direction of the light passing through the cell to an angle ϕ given by
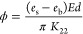
4where *E* is the applied in-plane electric field strength, *d* is the cell thickness, and  *K*_22_ is the twist elastic constant of the liquid crystal mixture. The results of the measurements of (*e*_s_ – *e*_b_) are presented in the results part of the paper.

### Out-of-plane Theoretical Model

As has been pointed out by Elston and Outram,^26^ the HAN out-of-plane electric field configuration is one possible method for measuring (*e*_s_ + *e*_b_) and is a system for which it is not possible to determine the difference in flexoelectric constants (*e*_s_ – *e*_b_). In order to model the out-of-plane electric field situation, we consider a 1D model of the HAN cell in Figure 7a, that is, of thickness *d* and with homeotropic (vertical) anchoring at the lower substrate and planar anchoring at the upper substrate, with variation in the *z* direction, perpendicular to the substrates. The free energy density of the liquid crystal layer under an applied electric field is the sum of the elastic energy density, dielectric energy density, and flexoelectric energy density given by
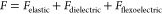
where
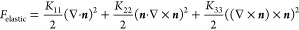

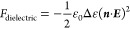

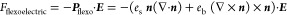


For particular values of the material parameters and applied voltage (see Table 2), we calculate the director configuration, ***n***(*z*) = (cos(θ(*z*)), 0, sin(θ(*z*))), and electric field, ***E***(*z*), by minimizing the free energy, that is, the integral of the free energy density over *z*, and solving Gauss’ law, respectively, subject to infinite planar and homeotropic at the upper and lower substrates.

**Table 2:** Parameter Values Used in Simulations for MDA-09-2329

parameter	value	source
*n*_e_	1.6152	Licristal data sheet
*n*_o_	1.4858	Licristal data sheet
ε_||_	25.6	Licristal data sheet
ε_⊥_	4.4	Licristal data sheet
Δε = ε_||_ – ε_⊥_	21.2	Licristal data sheet
*K*_11_	11.7 × 10^–12^ N	Licristal data sheet
*K*_22_	12.2 × 10^–12^ N	Licristal data sheet
*K*_33_	12.7 × 10^–12^ N	Licristal data sheet
λ	545 × 10^–9^ m	green bandpass filter
*d*	various	from experimental values in Table 3
*V*	various	from experimental values in Figure 10

The transmission *T* of light through the cell was calculated using the standard expression based on the retardance through a 1D birefringent layer
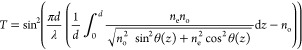
5where *n*_o_and *n*_e_ are the ordinary and extraordinary refractive indices respectively, and λ is the wavelength of the transmitted light.^35^

By solving for the director configuration for a range of values of (*e*_s_ + *e*_b_), we are able to fit the experimental transmission data to the theoretical values derived from eq 5 in order to find the estimated value of (*e*_s_ + *e*_b_).

### Determination of Dopant Flexoelectric Coefficients

The flexoelectric polarization of a liquid crystal mixture after dissolving a dopant within a host material can be written as
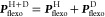
6where  and  are ***P***_flexo_ of the host and the dopant, respectively. If the host material and the flexoelectric dopant have opposite directions of the flexoelectric polarization, then their mixture has a reduced overall ***P***_flexo_ and possibly a change of the direction compared to the host material.^5,8^ As reported,^7^ at small concentrations of the dopant, flexoelectric splay *e*_s_ and bend *e*_b_ coefficients of the host/dopant liquid crystal mixture can be calculated using the linear approximation given by
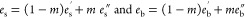
7where *m* is the dopant concentration (in the present study 5 wt %),  and  are the splay and bend flexoelectric coefficients of the host liquid crystal, and  and  are the splay and bend flexoelectric coefficients of the dopant. Such a linear dependence of (*e*_s_ – *e*_b_) as a function of the dopant concentration was obtained experimentally.^20^

### Measurements and Results

Low dopant concentration (5 wt %) was chosen in order to apply the linear approximation of the dependence of ***P***_flexo_ of the nematic liquid crystal host on the concentration of the dissolved dopant (see the Theoretical section). The difference of the flexoelectric coefficients *e*_s_ and *e*_b_, that is, (*e*_s_ – *e*_b_), was obtained from the electro-optic response of the HAN cell when applying in-plane dc electric field. The sum of *e*_s_ and *e*_b_, that is, (*e*_s_ + *e*_b_), was obtained by simulating the electro-optic effect, generated by application of out-of-plane dc electric field, and extract *e*_s_, *e*_b_, and their sum (*e*_s_ + *e*_b_) from the best fitting of the experimental curves.

For performing the measurements of the electro-optical response of the HAN cells under in-plane and out-of-plane switching, respectively, the cells were oriented between the crossed polarizers of the setup as depicted in Figure 7.

### Electro-Optic Response of HAN Cell under In-plane dc Electric Field—Measurement of the Difference (*e*_s_ – *e*_b_)

**Figure 9:**
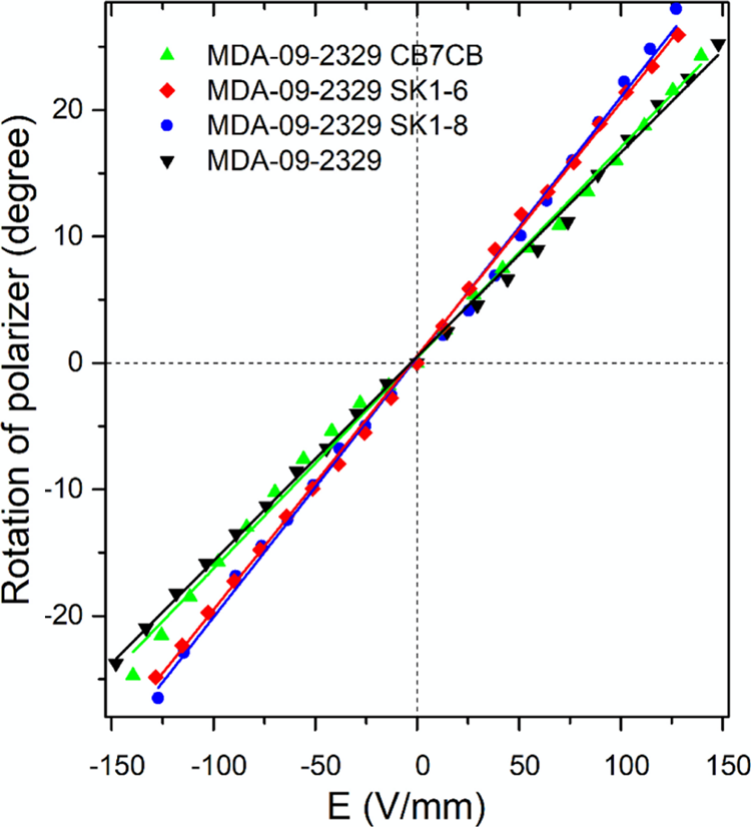
Resultant rotation of the polarizer needed to compensate for the director rotation, when an in-plane dc electric field is applied, and its linear dependence on the applied electric field.

For the measurements of *e*_s_ – *e*_b_, we chose the method described in the theoretical part.^31^ According to this method, in a cell with HAN configuration was applied in-plane dc electric field. The generated in-plane electric field was uniformly distributed over the area between the electrodes (i.e., over the electrode slit) and in a plane orthogonal to the plane of the HAN configuration, that is, in the plane of the substrates. The arrangement for this setup is schematically presented in Figure 7b. The cell optical axis in the field-off state is in the plane of the HAN director configuration and was oriented parallel to the light transmitting direction of one of the polarizers. In this position, the incoming light is therefore blocked by the crossed polarizers and the cell was in the dark state. The applied in-plane dc field induced twist along the substrates normal, which rotated the polarization plane of the incoming light by an angle ϕ, which can then be measured by rotating the polarizer in the direction of the switching of the liquid crystal molecules, clockwise or anticlockwise, until the experimental cell became dark again. The rotation angle of the polarizer therefore corresponds to the twist angle ϕ and is presented as a function of the applied electric field in Figure 9.

The available data in the literature for the value of (*e*_s_ – *e*_b_) for E7, measured by the same or different methods than the one used in this work, vary from 7.8 to 13 pC/m.^6,20,22,33,34^ From our measurements of (*e*_s_ – *e*_b_) for E7, performed in this work, we found that (*e*_s_ – *e*_b_) for E7 is 16.3 pC/m, which is in reasonable agreement with the values found by us before^6^ and by the other groups quoted above.

The results of the measurements for the host nematic MDA-09-2329 and corresponding mixtures containing CB7CB, SK 1–6, and SK 1–8 are shown in Table 3. As can be seen from Table 3, the calculated values of (*e*_s_ – *e*_b_) for the host nematic MDA-09-2329 and the corresponding mixtures with the dopants CB7CB, SK 1–6, and SK 1–8 were 36, 37.1, 43.3 and 44.4 pC/m, respectively. It should be pointed out that the value of (*e*_s_ – *e*_b_) found for the host nematic mixture MDA-09-2329 used in this work was 36.0 pC/m, which is 2 times larger than that of E7. This means that the host nematic mixture MDA-09-2329 has higher polarity than E7. The highest value of (*e*_s_ – *e*_b_) was measured for the mixture MDA-09-2329/SK 1–8, which was found to be 44.4 pC/m.

**Table 3:** Calculation of (*e*_s_ – *e*_b_) Using Equation 4

substance	cell thickness (μm)	ΔΦ/Δ*E* (deg/V/mm)	(*e*_s_ – *e*_b_)/*K*_22_ (C/Nm)	*K*_22_ (pN)	(*e*_s_ – *e*_b_) (pC/m)
E7	4.7	0.104	1.21	13.5^24^	16.3
MDA-09-2329	3.0	0.161	2.95	12.2	36.0
MDA-09-2329/CB7CB	3.0	0.167	3.04	12.2	37.1
	4.1	0.205	2.74	12.2	33.4
MDA-09-2329/SK 1–6	3.1	0.200	3.55	12.2	43.3
	4.4	0.280	3.49	12.2	42.6
MDA-09-2329/SK 1–8	3.1	0.206	3.64	12.2	44.4

Because the concentration of dopant dissolved in the nematic host was small, 5 wt %, we can use the linear approximation given by eq 7. We calculated the values of (*e*_s_ – *e*_b_) of the dopants CB7CB, SK 1–6, and SK 1–8 by eq 7 after rewriting it in the form
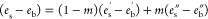
8where (*e*_s_ – *e*_b_), , and  are the differences in flexoelectric coefficients for the nematic mixture, host nematic mixture, and dopant, respectively, and m is the concentration of the dopant. The calculated values of (*e*_s_ – *e*_b_) of the dopants CB7CB, SK 1–6, and SK 1–8 are given in Table 4.

**Table 4:** Calculation Values of (*e*_s_ – *e*_b_) Using Equation 8

	CB7CB	SK 1–6	SK 1–8
*e*_s_ – *e*_b_	58 pC/m	182 pC/m	204 pC/m

As seen from Table 4, (*e*_s_ – *e*_b_) of the hockey stick dopants SK 1–6 and SK 1–8 is about 3 times larger than the one for CB7CB. Notice that the dimer CB7CB was reported recently in the literature for its giant flexoelectric response.^36^

### Electro-optic Response of HAN Cell under Out-of-plane dc Electric Field—Extracting *e*_s_, *e*_b_ and the Sum (*e*_s_ + *e*_b_) from the Best Fitting of the Electro-optic Response Curves

**Figure 10:**
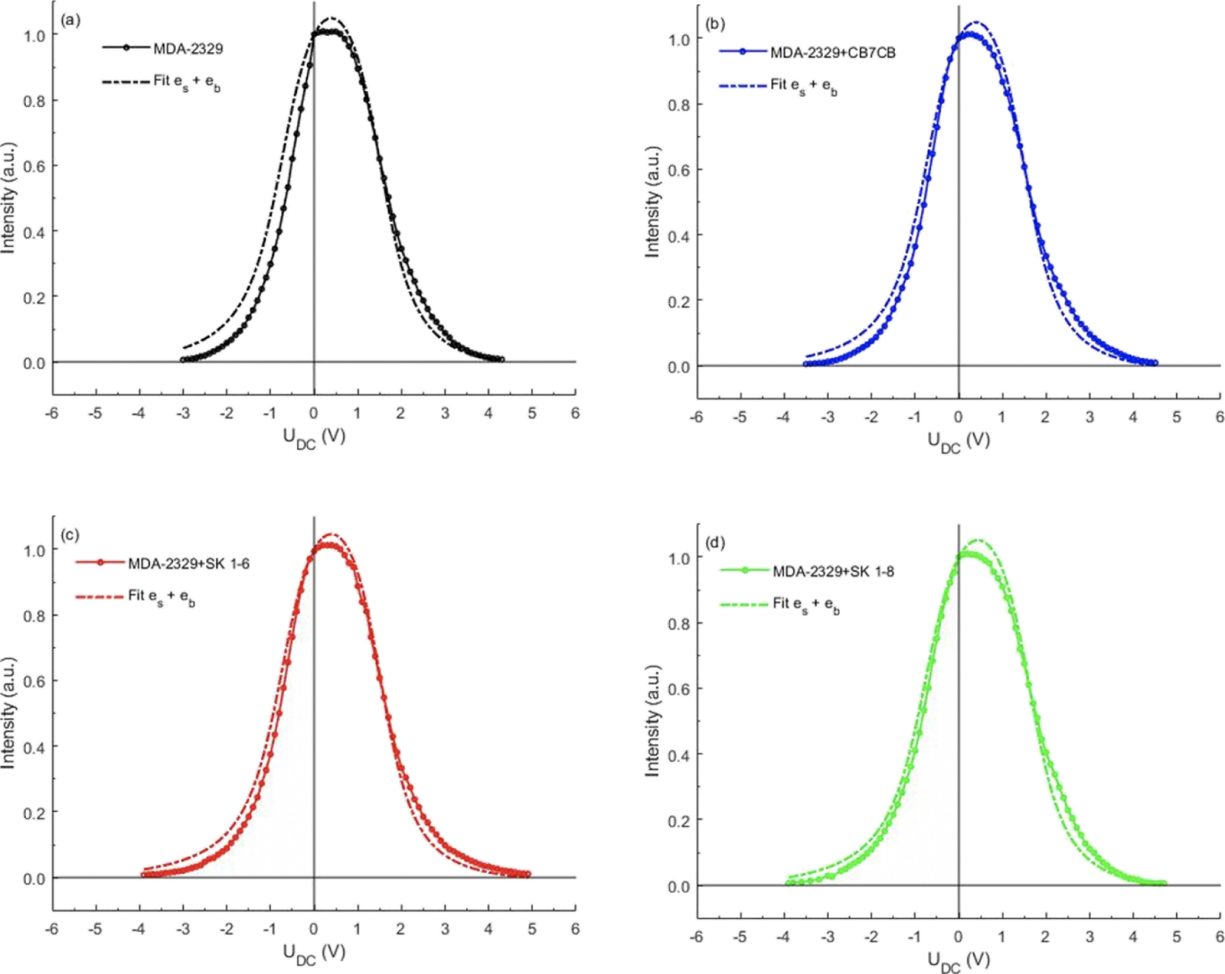
Intensity of transmitted green light λ = 545 nm through the HAN cells as a function of the applied out-of-plane dc voltage. Best fit transmission vs voltage curves for experimental (solid lines with circle symbols) and simulation (dash-dot line) for (a) host nematic mixture MDA-09-2329, (b) MDA-09-2329 with CB7CB dopant, (c) MDA-09-2329 with SK 1–6 dopant, and (d) MDA-09-2329 with SK 1–8 dopant. Fitting was undertaken by varying the average flexoelectric coefficient.

A dc electric field was applied across the liquid crystal layer by connecting the substrates’ electrodes as shown in Figure 2d, resulting in two areas of the cell available for measurement of the cell electro-optic response as a function of the applied field strength. An applied electric field led to a reorientation of the liquid crystal molecules to the vertical direction due to the dielectric coupling, which gives rise to an electro-optic response. To obtain the maximum transmitted light intensity in the field-off state, the experimental cell is oriented at 45° angle with respect to the direction of the polarizers in the experimental setup (the arrangement shown in Figure 7c). As expected, this electro-optic response was found to be nonsymmetric with respect to V = 0 (see Figure 10) due to the involvement of the flexoelectric coupling, which depends linearly on the electric field, that is, proportional to *E*, in contrast to the quadratic dependence of the dielectric effect, that is, proportional to *E*^2^.

As seen from the experimental curves shown in Figure 10, the electro-optic response of the cells containing the host liquid crystal mixture MDA-09-2329 and the mixtures of MDA-09-2329 with the dopants CB7CB, SK 1–6, and SK 1–8 are all asymmetric about V = 0. A purely dielectric effect would lead to symmetric reorientation to the vertical director orientation at high positive or negative voltages, which would result in a low transmission. However, we see that the flexoelectric polarization coupling with the applied dc voltage either enhances the dielectric effect leading to low transmission at lower magnitude voltage (for negative voltages) or detracts from the dielectric effect leading to low transmission only at higher magnitude voltages (for positive voltages). It is clear from the results presented in Figure 10 that the dopants increased the flexoelectric polarization of the host material and that the dopants SK 1–6 and SK 1–8 have quite a similar impact on the switching of the liquid crystal director, which is stronger than that of the dopants CB7CB.

### Fitting of the Electro-optical Response Curves

From the mathematical model described in the previous section, we were able to simulate the director distortion and thus the transmission through the cell, for various dc field strengths and any values of the flexoelectric splay and bend coefficients *e*_s_ and *e*_b_. By comparing the simulated transmission, as a function of voltage, to the experimental curves given in Figure 10, we were able to determine the value of *e*_s_ + *e*_b_ and, using the previous experimentally determined values of *e*_s_ – *e*_b_, therefore the values of *e*_s_ and *e*_b_ that best fit the data. The results of this fitting are given in Figure 10 and Table 5. Using the fitted values of the host and mixture flexoelectric coefficients in Table 5, the splay and bend coefficients for the dopants alone can be determined using eq 7. The corresponding values are presented in Table 6.

**Table 5:** Values of Splay and Bend Flexoelectric Coefficients that Allow a Best Fit between the Simulation and Experimental Results

	fitting (*e*_s_ + *e*_b_)			
	MDA-09-2329	MDA-09-2329 + CB7CB	MDA-09-2329 + SK 1–6	MDA-09-2329 + SK 1–6
*e*_s_	5.5 pC/m	4.55 pC/m	0.65 pC/m	3.2 pC/m
*e*_b_	–30.5	–32.55	–42.65	–41.2
*e*_s_ – *e*_b_	36	37.1	43.3	44.4
*e*_s_ + *e*_b_	–25.0	–28.0	–42.0	–38.0

**Table 6:** Calculated *e*_s_ and *e*_b_ Using Equation 7

	CB7CB (pC/m)	SK 1–6 (pC/m)	SK 1–8 (pC/m)
*e*_s_	–13.5	–91.5	–40.5
*e*_b_	71.5	273.5	244.5
*e*_s_ – *e*_b_	58	182	204
*e*_s_ + *e*_b_	–85	–365	–285

It was also observed experimentally that a low magnitude negative dc voltage induced a small twist deformation, of about 3° (due to an out-of-plane switching of the molecules). It is proposed that this twisting of the HAN configuration leads to a high intensity of transmitted light to be maintained up to the negative dc threshold voltage.

## Discussion

During the last decade much effort has been put into the investigation and tailoring of the magnitude of ***P***_flexo_ in nematic liquid crystals with the aim of their use in liquid crystal device applications such as ultrafast (μs) liquid crystal devices.^12,13^ There were efforts, both theoretical and experimental, to find those parameters of the liquid crystal, in general, and of the liquid crystal molecular structure, in particular, which are playing the key role in determining liquid crystal flexoelectric polarizability.

The idea of enhancing the flexoelectricity of the liquid crystal by dopants with pronounced molecular shape polarity was first reported already in 1998.^5^ By dissolving such dopants, which may or may not possess a nematic phase, in an appropriate nematic host material, a liquid crystal mixture with a broad temperature interval of the nematic phase and enhanced flexoelectric polarizability could be obtained.

In our previous work, we used an azo dye [4-hexyloxy-(4′hexyl)azobenzene] as a dopant for increasing the ***P***_flexo_ of a nematic mixture.^6^ When the azo dye was dissolved by 5 wt % in the nematic host E7, the magnitude of (*e*_s_ – *e*_b_)/*K* of E7 slightly increased from −1.7 to −1.9 C/Nm. However, after UV illumination of the sample for 60 s, (*e*_s_ – *e*_b_)/*K* became −2.6 C/Nm, that is, increased by 40%, due to the generation of the *cis*-isomer, which has bend shape, thus demonstrating the importance of molecular shape anisotropy.^3,6^ Another experimental result confirming this conclusion was found when a bend-shape dopant (B10), whose molecular structure contains five phenyl rings connected by a mixture of ester and imine linking groups, was dissolved by 5 wt % in the nematic mixture E7, changed the value of (*e*_s_ – *e*_b_)/*K* from 1 to −1.3 C/Nm, increasing the magnitude and reversing its sign.^5^ This means that (*e*_s_ – *e*_b_)/*K* effectively increased by 2.3 C/Nm. Similar sign reversal of (*e*_s_ – *e*_b_)/*K* was also reported after doping the nematic host [1,1′-biphenyl]-4-yl 4-(undecyloxy)benzoate, the molecules of which have rodlike shape, with the bent core liquid crystal BC120.^8^ Moreover, an almost linear increase of (*e*_s_ – *e*_b_)/*K* from 2.18 to 3.6 C/Nm was observed when the concentration of the dopant BC120 increased from 4.8 to 7 M %.

In several studies, the correlations between flexoelectric polarizability of doped nematic liquid crystals and dopant molecular parameters, such as molecular length *a*, bend angle θ_ο_, and the components of the molecular net dipole moment μ_resultant_ (*p*_||_ and *p*_⊥_, according to the Helfrich notification) were considered.^19−24^ The results of these studies suggest that highly polar molecules with a very pronounced molecular shape anisotropy, such as wedge-, highly kinked-, or banana-shape, as well as a nematic host material with high polarity are required to induce a large flexoelectric ***P***_flexo_. However, so far, the reported results are either contradictory or there are insufficient numbers to enable a clear and unambiguous picture of these correlations. It is not yet possible to propose appropriate combinations of dopants that can serve as a guideline for design, synthesis, and preparation of liquid crystal mixtures with enhanced flexoelectric polarizability.

The goal of our present work was to study and compare the change in ***P***_flexo_ for a nematic host induced by two different kinds of flexoelectric dopants with molecular shape anisotropy such as bend and hockey stick, respectively. All dopants possess liquid crystalline phase in their phase sequencies. Moreover, in one of them, SK 1–8, the nematic phase is cybotactic, that is, it contains molecular clusters (cybotactic groups) in which the molecules have a smectic-like order.^29,30^ It should be noted that the formation of clusters in the nematic phase depends not only on the underlaying smectic phase but also on the molecular dipole moment and bend shape.

To perform a study on the flexoelectric properties of the three nematic liquid crystals, the dimer CB7CB and hockey stick liquid crystals, SK 1–6 and 1–8, would be difficult due to high-temperature nematic phases of the last two dopants, which usually are either difficult to obtain or can destroy the HAN configuration. Therefore, these materials were dissolved as dopants in a nematic host MDA-09-2329, highly polar liquid crystal mixture, and was used as a host material. These dopants were dissolved in the host material in small concentration (5 wt %) so that the linear approximation given by eq 7 could be applied for obtaining the flexoelectric coefficients *e*_s_ and *e*_b_ of the pure dopant materials. The low concentration of the dopant dissolved in the host liquid crystal mixture is assumed not to affect notably the physical properties of the nematic host.

According to the existing theories, the difference between *e*_s_ and *e*_b_, that is, (*e*_s_ – *e*_b_), is only related to the dipolar flexoelectric effect, whereas their sum (*e*_s_ + *e*_b_) is related to both dipolar and quadrupolar flexoelectric effects. As reported here, the dopants have negative sign of (*e*_s_ + *e*_b_) and usually is larger in absolute value than (*e*_s_ – *e*_b_), thus being in accordance with the theoretical model of flexoelectric effect.^37^

### Nematic Host MDA-09-2329

The nematic host MDA-09-2329 has Δε = 21.2 and thus is highly polar. The measured (*e*_s_ – *e*_b_)/*K* of the host nematic liquid crystal was found to be 2.95 C/Nm, which results in (*e*_s_ – *e*_b_) = 36 pC/m, and from the fitting routine, it was found that (*e*_s_ + *e*_b_) = −25 pC/m was obtained. The high values of (*e*_s_ – *e*_b_) and (*e*_s_ + *e*_b_) indicate that both dipolar and quadrupolar effects contribute to the flexoelectric polarizability of the host material, and the dipolar contribution is stronger due to the strong polarity of the constituent liquid crystal molecules of the nematic host. As already mentioned, compared with the host material E7, which has (*e*_s_ – *e*_b_) = 16.3 pC/m, the host nematic MDA-09-2329 has 2 times stronger (*e*_s_ – *e*_b_). Therefore, the nematic liquid crystal MDA-09-2329 seems to be a good candidate as a host material for preparation of flexoelectric mixtures.

### Dopant CB7CB

In previous work, it was proposed to use dimeric liquid crystals as materials with high flexoelectric polarizability.^7,19^ The dimeric liquid crystal CB7CB was studied in this work as a dopant for enhancement of ***P***_flexo_ of the host nematic MDA-09-2329. The dimer did indeed exhibit a strong flexoelectric polarizability. By means of the previously described method,^18^ it was derived for CB7CB, (*e*_s_ – *e*_b_)/*K* = 7.34 C/Nm from the flexoelectro-optic response in short pitch cholesteric mixture based on CB7CB (93.19 wt %) and aligned in UHL texture.^36^ Considering that K = (*K*_11_ + *K*_33_)/^2^ for CB7CB is 4 pN^28^, we obtain (*e*_s_ – *e*_b_) = 29.4 pC/m. In the present work, according to eq 7, we estimated for CB7CB in our cells with HAN configuration (*e*_s_ – *e*_b_) = 58 pC/m, that is, it is almost twice higher than the one measured previously.^36^ The possible reason for this, we believe, might be that the measurement methods were different. Moreover, as can be seen from Table 3, when the strength of the elastic deformation of the HAN configuration is increased, by decreasing the cell gap thickness, the value of (*e*_s_ – *e*_b_) increased, thus justifying the importance of HAN configuration profile on ***P***_flexo_ due to the doping effect of CB7CB. Moreover, the HAN configuration may also introduce an additional increase of the concentration of the bend conformations of CB7CB molecules and thus increase, in addition, the dipolar contribution to the ***P***_flexo_ of the HAN cell.

Another investigation of the flexoelectric properties of a nematic mixture doped with the dimeric dopant CB7CB shows the importance of the host material.^38^ In this work, the value of (*e*_s_ – *e*_b_) for the nematic mixture ZLI4330/CB7CB (70/30 wt %) was investigated with host ZLI4330, having Δε = −1.7, in a HAN cell with a gap thickness of 7 μm. The obtained value of (*e*_s_ – *e*_b_) for this nematic mixture was 6.96 pC/m. For the investigation in the present work, the nematic mixture MDA-09-2329/CB7CB (95/5 wt %), we found (*e*_s_ – *e*_b_) = 37.1 pC/m, that is almost 6 times stronger than the latter case, although the concentration of the dopant CB7CB was 6 times less. Two reasons are assumed to be responsible for such a big difference:

(a) The host nematic materials ZLI4330 and MDA-09-2329 have very different polarities. The polarity of ZLI4330 is much lower than that of MDA-09-2329, thus indicating that the polarity of the host material as well as the interactions between host and dopant materials have an important impact on ***P***_flexo_.

(b) The thickness of the HAN cell gap containing the mixture ZLI4330/CB7CB is more than 2 times larger than the one of the HAN containing MDA-09-2329/CB7CB, and thus the strength of the elastic deformation in the HAN configuration in the cell containing ZLI4330/CB7CB will be weaker and consequently the flexoelectric polarization lower.

According to the theory of flexoelectricity in liquid crystals,^39^ the *e*_s_ coefficient of CB7CB should be zero. However, as seen from the Table 6, *e*_s_ of the dimer CB7CB dissolved in the host nematic liquid crystal has non-zero value, probably due to the HAN configuration of the liquid crystal mixture in the cell as well as being due to the influence of the host material on the CB7CB conformations. The sum of the flexoelectric constant of CB7CB, (*e*_s_ + *e*_b_), reflecting the effect of quadrupolar contribution to ***P***_flexo_ of its 5 wt % mixture with the polar nematic host material MDA-09-2329, has a value of −28 pC/m, similar to the one of the host material MDA-09-2329, which is −25 pC/m.

### Dopants SK 1–6 and 1–8

**Figure 11:**
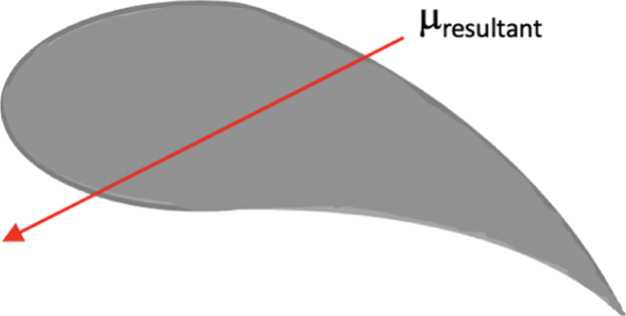
Idealized picture of the molecule of flexoelectric dopant with bend/pear shape and direction of molecular net dipole moment inclined with respect to the molecule as shown in the picture. This molecular form of the dopant and orientation of the molecular net dipole moment are assumed to be suitable for both bend and splay deformations, giving rise to ***P***_flexo_ in a nematic host, as discussed by Dahl.^40^

The hockey stick dopants SK 1–6 and SK 1–8 were of particular interest for our study. According to the theoretical model of Dahl,^40^ liquid crystals with hybrid bend/pear shaped molecules and for a molecular net dipole moment inclined with respect to the molecule long arm in the way shown in Figure 11, or those that contain dopants with these properties, are expected to exhibit high ***P***_flexo_. According to this model, molecules with such a hybrid bend/pear molecular shape fit well to both splay and bend elastic deformations of the liquid crystal and are therefore appropriate for enhancement of the flexoelectric properties of the liquid crystal. As can be seen from their DFT optimized structure, both flexoelectric dopants, SK 1–6 and SK 1–8, studied in this work have very similar molecular shapes as the one shown in Figure 11. We found, using eq 8, that the dopants SK 1–6 and SK 1–8 have values for (*e*_s_ – *e*_b_) of 0.182 and 0.204 nC/m, respectively. These values are more than 3 times higher than that obtained for CB7CB. Notice that the dimer CB7CB was reported in the literature in 2017 as the liquid crystal material exhibiting the highest flexoelectro-optic effect due to the large ***P***_flexo_.^36^

The quadrupole effect is much stronger in the case of the hockey dopants SK 1–6 and SK 1–8 than in the case of bend-shape dopant CB7CB. When dissolved 5 wt % in the host nematic MDA-09-2329, (*e*_s_ + *e*_b_) is −42 pC/m for SK 1–6 and −38 pC/m for SK 1–8. Hence, the estimated sum (*e*_s_ + *e*_b_) for pure SK 1–6 and 1–8 is found −365 and −285 pC/m, respectively. The possible reason for this difference might be related to the different orientation and magnitude of the molecular net dipole moment as well as to the length of the dopant molecules. As already mentioned, the molecular net dipole of SK 1–6 is larger than the one of SK 1–8, and it is more inclined with respect to the long molecular arm (cf. Table 1). The orthogonal component of the molecular net dipole moment of SK 1–6 is also larger. The presence of cybotactic groups in the nematic phase of SK 1–8 may also lead to redistribution of the dipolar and quadrupolar contributions in ***P***_flexo_ of the liquid crystal mixture containing one of these dopants. In accordance with the accepted opinion in the literature and the available data about (*e*_s_ + *e*_b_), (*e*_s_ + *e*_b_) has minus sign and much higher value than (*e*_s_ – *e*_b_).^37,41^

There are several theories and models attempting to explain the correlations between the molecular structural parameters and the flexoelectric properties of the liquid crystals which are widely spread in the literature, but none of them gives a clear picture of these correlations. Moreover, their conclusions are in some cases even in contradiction with the reported experimental results. It is even less clear what the impact of the host/dopant interactions has on the ***P***_flexo_ of the nematic liquid crystal mixture containing a dopant. Of special interest, however, seems to be the case of host nematic liquid crystal with high polarity mixed with a high polar dopant with a mixed bend/pear shape, fitting both splay and bend deformations of the liquid crystal host. Such a flexoelectric nematic mixture is expected to possess high ***P***_flexo_ being of strong interest for device applications.

## Conclusions

In our previous studies, we proved that the change of the molecular shape from linear (*trans*-isomer) to bend shape (*cis*-isomer) during photoisomerization plays a major role in the increase of ***P***_flexo_ of photoresponsive nematics, whereas the change in the magnitude of the molecular net dipole moment is of minor importance.^3,4^ As proved by other researchers, ***P***_flexo_ is sensitive to molecule characteristics of the liquid crystals as well as to those of the dopants dissolved therein, such as molecular shape anisotropy and molecular length, but it is rather insensitive to the magnitude of the molecular net dipole moment.^19−22^ It is important to point out that all three dopants investigated in this work have the nematic phase in their phase sequences. The bend-shape dopant CB7CB is a flexible dimer. It has flexible aliphatic central link and therefore being capable of more easily adopting a splay elastic deformation of the liquid crystal than dopants with a more rigid bend shape.^19^ The hockey stick dopants SK 1–6 and SK 1–8, studied in this work, however, can much easily adopt both splay and bend elastic deformations of the liquid crystal due to their shape. Moreover, these dopants have a large molecular length, and their molecular net dipole moments are inclined with respect to their long arm, thus having large longitudinal and transfer dipole components. The magnitudes of (*e*_s_ – *e*_b_) and (*e*_s_ + *e*_b_) reported here for these two hockey stick dopants are with the highest value reported so far, (*e*_s_ – *e*_b_) = 182 pC/m and (*e*_s_ + *e*_b_) = −365 pC/m for SK 1–6 and (*e*_s_ – *e*_b_) = 204 pC/m and (*e*_s_ + *e*_b_) = −285 pC/m for SK 1–8. It should be also pointed out that the host nematic material MDA-09-2329 is highly polar, with Δε = 21.2, and (*e*_s_ – *e*_b_) = 36 pC/m and (*e*_s_ – *e*_b_) = −25 pC/m.

In conclusion, dopants with a hockey stick molecular shape as well as with large molecular net dipole, that is, being highly polar, which transfers and longitudinal components are large too, seems to be appropriate as flexoelectric dopants. Flexoelectric liquid crystal mixtures containing such dopants would substantially improve the performance of the liquid crystal, whose operation is based on the flexoelectric coupling.^42^
